# EFE-8c4, a polyamine from *Elaeagnus multiflora*, protects neuronal cells by regulating oxidative stress and apoptotic pathways

**DOI:** 10.3389/fnins.2026.1868462

**Published:** 2026-07-02

**Authors:** Da-In Choi, HuiJun Lee, Chul-Yung Choi

**Affiliations:** 1 Well-Aging Medicare and City G-LAMP Project Group, Chosun University, Gwangju, Republic of Korea; 2 Department of Integrative Biological Sciences and BK21 FOUR Educational Research Group for Age-Associated Disorder Control Technology, Chosun University, Gwangju, Republic of Korea; 3 Department of Biomedical Science, Chosun University, Gwangju, Republic of Korea

**Keywords:** apoptosis, corticosterone, *Elaeagnus multiflora*, neuroprotection, oxidative stress, polyamine compounds, SH-SY5Y cells

## Abstract

Neurological disorders, including depression, cognitive impairment, and neurodegenerative diseases, are closely associated with oxidative stress, apoptotic neuronal loss, and impaired neuronal differentiation. Polyamine derivatives from natural products have emerged as potential neuroprotective agents, although their mechanisms remain incompletely understood. In this study, three polyamine compounds isolated from *Elaeagnus multiflora* fruit were evaluated in SH-SY5Y cell models of oxidative and glucocorticoid-induced stress. Among these, EFE-8c4 exhibited the most pronounced neuroprotective activity, significantly restoring cell viability under corticosterone-induced stress and attenuating oxidative damage induced by hydrogen peroxide. Mechanistically, EFE-8c4 modulated apoptotic signaling by increasing Bcl-2 expression while suppressing Bax, caspase-8, and p53 activation, thereby restoring the balance between pro- and anti-apoptotic pathways. In addition, EFE-8c4 reduced intracellular reactive oxygen species accumulation and enhanced the expression of PCNA and βIII-tubulin, indicating improved cell survival capacity and neuronal phenotype maintenance. Furthermore, EFE-8c4 partially reduced apoptotic cell populations under corticosterone exposure. Collectively, these findings demonstrate that EFE-8c4 exerts multi-target neuroprotective effects through coordinated regulation of apoptosis, oxidative stress, and neuronal differentiation-related pathways, highlighting its potential as a candidate for the treatment of oxidative stress-associated neurological disorders.

## 1 Introduction

Neurological disorders, including major depressive disorder, cognitive impairment, Parkinson’s disease (PD), and Alzheimer’s disease (AD), represent a substantial and increasing global health burden, affecting more than 3 billion individuals worldwide and constituting the leading cause of disability-adjusted life years globally (GBD 2021 Nervous System Disorders Collaborators, 2024). A fundamental pathological feature shared by these conditions is the disruption of the delicate imbalance between neuronal survival, death, and regenerative capacity, leading to progressive neuronal loss and functional decline (Dugger and Dickson, 2017; Bredesen et al., 2006). Oxidative stress, mitochondrial dysfunction, and aberrant apoptotic signaling are key molecular mechanisms underlying neurodegeneration (Lin and Beal, 2006; Barnham et al., 2004; Gandhi and Abramov, 2012).

The human neuroblastoma SH-SY5Y cell line has emerged as a widely utilized *in vitro* model for investigating neuroprotective compounds and elucidating mechanisms of neuronal survival and death (Shipley et al., 2016; Targett et al., 2024). When differentiated with retinoic acid (RA), SH-SY5Y cells acquire mature neuronal characteristics including neurite outgrowth and expression of neuronal markers such as βIII-tubulin, and enhanced susceptibility to neurotoxic insults, thereby recapitulating key features of human neurons. The differentiation protocol involves sequential serum reduction combined with RA treatment, which promotes neuronal phenotype while selecting against epithelial-like cells (Park et al., 2024).

Polyamines including putrescine, spermidine, and spermine are ubiquitous polycationic molecules essential for cell growth, differentiation, and survival. In the nervous system, polyamines play critical roles in neuronal development, synaptic plasticity, and neuroprotection (Pegg, 2016; Igarashi and Kashiwagi, 2010). Polyamine homeostasis is tightly regulated, and its disruption has been associated with aging and various pathological states (Minois et al., 2011). Spermidine has been shown to induce autophagy, reduce oxidative stress, and extend lifespan in model organisms (Madeo et al., 2018; Eisenberg et al., 2009). Spermine modulates NMDA receptor function and ion channel activity, protecting against excitotoxicity (Liu et al., 2022). Putrescine serves as a precursor for higher polyamines and participates in cellular stress responses (Pegg, 2009). Importantly, dysregulation of polyamine metabolism has been implicated in neurodegenerative diseases, and polyamine supplementation has demonstrated neuroprotective effects in various experimental models (Kalecký and Bottiglieri, 2022; Vrijsen et al., 2023).

*Elaeagnus multiflora* Thunb. (Elaeagnaceae), commonly known as cherry elaeagnus or goumi, is a deciduous shrub native to East Asia. Its fruits have been traditionally used in Korean and Chinese medicine for their purported health benefits, including anti-inflammatory, antioxidant, and immunomodulatory properties. Phytochemical investigations have revealed that Elaeagnus species contain diverse bioactive compounds including flavonoids, phenolic acids, terpenoids, and nitrogen-containing compounds. However, the neuroprotective potential of polyamine compounds from *E. multiflora* has not been fully elucidated.

Corticosterone (CORT) is widely used to induce stress-related neuronal damage in cellular models, mimicking glucocorticoid-induced neurotoxicity (Sapolsky, 2000). Chronic elevation of glucocorticoids leads to neuronal atrophy, impaired neurogenesis, and increased vulnerability to oxidative stress, particularly in hippocampal neurons (McEwen et al., 2016). CORT-induced neurotoxicity involves multiple mechanisms, including mitochondrial dysfunction, oxidative stress, and activation of apoptotic pathways.

In parallel, hydrogen peroxide (H₂O_2_) is a well-established inducer of oxidative stress, leading to reactive oxygen species (ROS) accumulation and apoptotic cell death (Uttara et al., 2009; Halliwell, 2006). Prolonged or dysregulated stress exposure has been shown to disrupt neuronal structural plasticity and functional adaptation, thereby contributing to the progression from physiological stress responses to pathological brain conditions (Joëls et al., 2018; De Kloet et al., 2005).

The present study aimed to isolate and characterize polyamine compounds from *E. multiflora* fruit extract and to evaluate their neuroprotective effects against CORT- and H₂O_2_-induced neurotoxicity in differentiated SH-SY5Y cells. We hypothesized that these polyamine compounds exert neuroprotective effects by modulating apoptotic signaling and oxidative stress pathways.

## 2 Materials and methods

### 2.1 Plant material and compounds

Three polyamine compounds (EFE-8c1, EFE-8a2a, and EFE-8c4) were isolated from an aqueous extract of *Elaeagnus multiflora* Thunb. fruits and provided by the Jeonnam Institute of Natural Resources Research (Jeollanam-do, Republic of Korea). Voucher specimens were deposited at the Jeonnam Institute of Natural Resources Research herbarium. All three compounds share the molecular formula C_34_H_37_N_3_O_6_ (MW 583.7 g/mol). Stock solutions were prepared at 10 mg/mL in dimethyl sulfoxide (DMSO) and stored at −20 °C.

### 2.2 Cell culture and differentiation

Human neuroblastoma SH-SY5Y cells (ATCC, Manassas, VA, USA) were generally maintained in Dulbecco’s Modified Eagle’s Medium/Nutrient Mixture F-12 (DMEM/F12, 1:1) supplemented with 10% fetal bovine serum (FBS; Gibco, Grand Island, NY, USA, Cat. No. 16000-044), 100 U/mL penicillin and 100 μg/mL streptomycin (P/S; Gibco, Cat. No. 15140-122) at 37 °C in a humidified atmosphere containing 5% CO₂. Unless otherwise indicated, SH-SY5Y cells were maintained and used in their undifferentiated state under these culture conditions. For neuronal differentiation experiments only, SH-SY5Y cells were expanded before differentiation in Minimum Essential Medium (MEM) supplemented with 15% FBS (Gibco, Cat. No. 16000-044), 1% P/S (Gibco, Cat. No. 15140-122), and 2 mM L-glutamine (Gibco, Cat. No. 25030-081). Neuronal differentiation was induced by replacing the growth medium with differentiation medium containing reduced serum (2.5% FBS) and 10 μM all-trans retinoic acid (RA; Sigma-Aldrich, St. Louis, MO, USA, Cat. No. R2625-50MG), as summarized in Table 1, Shipley et al. (2016), and Targett et al. (2024). The differentiation medium was prepared by adding 100 μL of RA stock solution to 50 mL of medium, corresponding to a final RA concentration of 10 μM. The medium was replaced every 1–2 days during the differentiation period. Neuronal differentiation was confirmed by morphological assessment of neurite outgrowth and immunofluorescence staining for βIII-tubulin (Park et al., 2024).

**Table 1:** Composition of growth and differentiation media for SH-SY5Y human neuroblastoma cells.

Medium	Component	Preparation
Growth	MEM	Base medium
	FBS	Add to 15% (v/v)
	Pen/Strep	1:100 dilution
	L-glutamine	2 mM final
Differentiation	MEM	Base medium
	FBS	Add to 2.5% (v/v)
	Pen/Strep	1:100 dilution
	L-glutamine	2 mM final
	RA	Add from stock to 10 μM final

The differentiation medium was supplemented with retinoic acid (RA) to induce neuronal differentiation.

### 2.3 Cytotoxicity and neuroprotection assays

For cytotoxicity assessment, cells were treated with increasing concentrations (0–100 μg/mL) of EFE-8c1, EFE-8a2a, or EFE-8c4 for 24 h under normal culture conditions. Control cells were treated with vehicle (dimethyl sulfoxide, DMSO; Sigma-Aldrich, St. Louis, MO, USA, Cat. No. D4540) at a final concentration not exceeding 0.1%. Cell viability was expressed as a percentage relative to untreated control cells. For neuroprotection assays, cells were pretreated with each compound for 1 h, followed by co-treatment with corticosterone (CORT; Sigma-Aldrich, St. Louis, MO, USA, Cat. No. 27840-100MG; 100 μM) or hydrogen peroxide (H_2_O_2_; Sigma-Aldrich, St. Louis, MO, USA, Cat. No. H1009-100ML; 250 μM) for 24 h. CORT was dissolved in ethanol (final ethanol concentration ≤ 0.1%) and used as a model of glucocorticoid-induced neuronal stress (Sapolsky, 2000; McEwen et al., 2016). After treatment, MTS reagent (CellTiter 96® AQueous One Solution Cell Proliferation Assay; Promega, Madison, WI, USA, Cat. No. G3582) was added to each well and incubated according to the manufacturer’s protocol. Absorbance was measured at 490 nm using a microplate reader, and cell viability was calculated relative to the control group. All experiments were performed in triplicate, and data are presented as mean ± SEM.

### 2.4 Western blot analysis

Following treatment, cells were washed with ice-cold phosphate-buffered saline (PBS) and lysed in radioimmunoprecipitation assay (RIPA) buffer (50 mM Tris–HCl, pH 7.4, 150 mM NaCl, 1% NP-40, 0.5% sodium deoxycholate, and 0.1% SDS) supplemented with protease inhibitor cocktail tablets (cOmplete™, EDTA-free Protease Inhibitor Cocktail; Roche Diagnostics, Mannheim, Germany, Cat. No. 11873580001). Protein concentrations were determined using a bicinchoninic acid (BCA) protein assay kit (Pierce™ BCA Protein Assay Kit; Thermo Fisher Scientific, Rockford, IL, USA, Cat. No. 23225). Equal amounts of protein (30 μg per lane) were separated by 8–12% SDS-polyacrylamide gel electrophoresis (SDS-PAGE) and transferred onto polyvinylidene fluoride (PVDF) membranes (Millipore, Burlington, MA, USA, Cat. No. IPVH85R). Membranes were blocked with 5% non-fat dry milk in Tris-buffered saline containing 0.1% Tween-20 (TBST) for 1 h at room temperature and incubated overnight at 4 °C with the following primary antibodies: anti-PCNA (1:1000; Cell Signaling Technology, Danvers, MA, USA, Cat. No. 13110), anti-Bcl-2 (1:1000; Cell Signaling Technology, Cat. No. 3498), anti-Bax (1:1000; Cell Signaling Technology, Cat. No. 2772), anti-caspase-8 (1:1000; Abcam, Cambridge, UK, Cat. No. ab108333), anti-p53 (1:1000; Cell Signaling Technology, Cat. No. 9282), anti-GAPDH (1:5000; Cell Signaling Technology, Cat. No. 2118). After washing with TBST, membranes were incubated with HRP-conjugated secondary antibodies (1:5000; Cell Signaling Technology, Danvers, MA, USA) for 1 h at room temperature. Protein bands were visualized using enhanced chemiluminescence reagent (SuperSignal™ West Pico PLUS Chemiluminescent Substrate; Thermo Fisher Scientific, Waltham, MA, USA, Cat. No. 34580) and quantified by densitometric analysis using ImageJ software (National Institutes of Health, Bethesda, MD, USA). GAPDH was used as loading control (Olufunmilayo et al., 2023).

### 2.5 Immunocytochemistry

Differentiated SH-SY5Y cells were grown on glass coverslips and treated as described above, then fixed with 4% paraformaldehyde in phosphate-buffered saline (PBS) for 15 min at room temperature. After permeabilization with 0.1% Triton X-100 for 10 min and blocking with 5% normal goat serum for 1 h, cells were incubated overnight at 4 °C with the following primary antibodies: anti-PCNA (1:200; Cell Signaling Technology, Danvers, MA, USA, Cat. No. 13110) and anti-βIII-tubulin (1:500; Cell Signaling Technology, Danvers, MA, USA, Cat. No. 5568). After washing with PBS, cells were incubated with Alexa Fluor® 488-conjugated secondary antibodies (1:500; Cell Signaling Technology, Danvers, MA, USA, Cat. No. 4412) for 1 h at room temperature in the dark. Nuclei were counterstained with DAPI-containing mounting medium (Fluoroshield™ with DAPI; Abcam, Cambridge, UK, Cat. No. ab104139). Fluorescence images were acquired using an inverted fluorescence microscope (NIB610FL; Ningbo Yongxin Optics Co., Ltd., Ningbo, China) at 20 × and 40 × magnification. Fluorescence intensity was quantified using ImageJ software (National Institutes of Health, Bethesda, MD, USA) (Olufunmilayo et al., 2023; Angelova and Abramov, 2016).

### 2.6 Flow cytometry

Apoptosis was quantified using an Annexin V-FITC/PI Apoptosis Detection Kit (BD Biosciences, San Jose, CA, USA; Cat. No. 556547) according to the manufacturer’s protocol. Undifferentiated SH-SY5Y cells were treated with corticosterone (CORT, 200 μM, 24 h) in the presence or absence of EFE-8c4 (10 μg/mL). To ensure sufficient induction of apoptotic cell death for flow cytometric analysis, a higher concentration of CORT was used in this experiment. After treatment, cells were gently harvested by trypsinization, washed twice with cold PBS, and resuspended in Annexin V binding buffer at a density of 1 × 10^6^ cells/mL. Cells were stained with Annexin V-FITC (5 μL) and propidium iodide (PI; 5 μL) for 15 min at room temperature in the dark (Angelova and Abramov, 2016).

### 2.7 Reactive oxygen species detection

Intracellular reactive oxygen species (ROS) levels were measured using the 2′,7′-dichlorodihydrofluorescein diacetate (H2DCFDA; Invitrogen™, Thermo Fisher Scientific, Eugene, OR, USA, Cat. No. D399) assay. Differentiated SH-SY5Y cells were seeded on glass coverslips and treated with H₂O_2_ (250 μM, 24 h) in the presence or absence of EFE-8c4 (5 and 10 μg/mL). Cells were then incubated with H2DCFDA (10 μM) in serum-free medium for 30 min at 37 °C, washed with phosphate-buffered saline (PBS), and counterstained with DAPI-containing mounting medium (Fluoroshield™ with DAPI; Abcam, Cambridge, UK, Cat. No. ab104139). Fluorescence images were acquired using a fluorescence microscope (NIB610FL; Ningbo Yongxin Optics Co., Ltd., Ningbo, China) at 20× and 40× magnification. DCF fluorescence intensity (green channel) was quantified using ImageJ software (National Institutes of Health, Bethesda, MD, USA) (Olufunmilayo et al., 2023; Angelova and Abramov, 2016).

### 2.8 Statistical analysis

All experiments were performed in at least three independent replicates. Data are presented as mean ± SEM. Statistical comparisons were performed using one-way ANOVA followed by Dunnett’s *post hoc* test for multiple comparisons against the relevant control or stress-treated group, or Student’s t-test for two-group comparisons, using JASP software (version 0.19.3; University of Amsterdam, Amsterdam, The Netherlands). Statistical significance: *p < 0.05, **p < 0.01, ***p < 0.001 vs. control; #*p* < 0.05, ##*p* < 0.01, ###*p* < 0.001 vs. CORT-treated group or H_2_O_2_-treated group.

## 3 Results

### 3.1 Cytotoxicity of EFE-8c1, EFE-8a2a, and EFE-8c4 in differentiated SH-SY5Y cells

**Figure 1:**
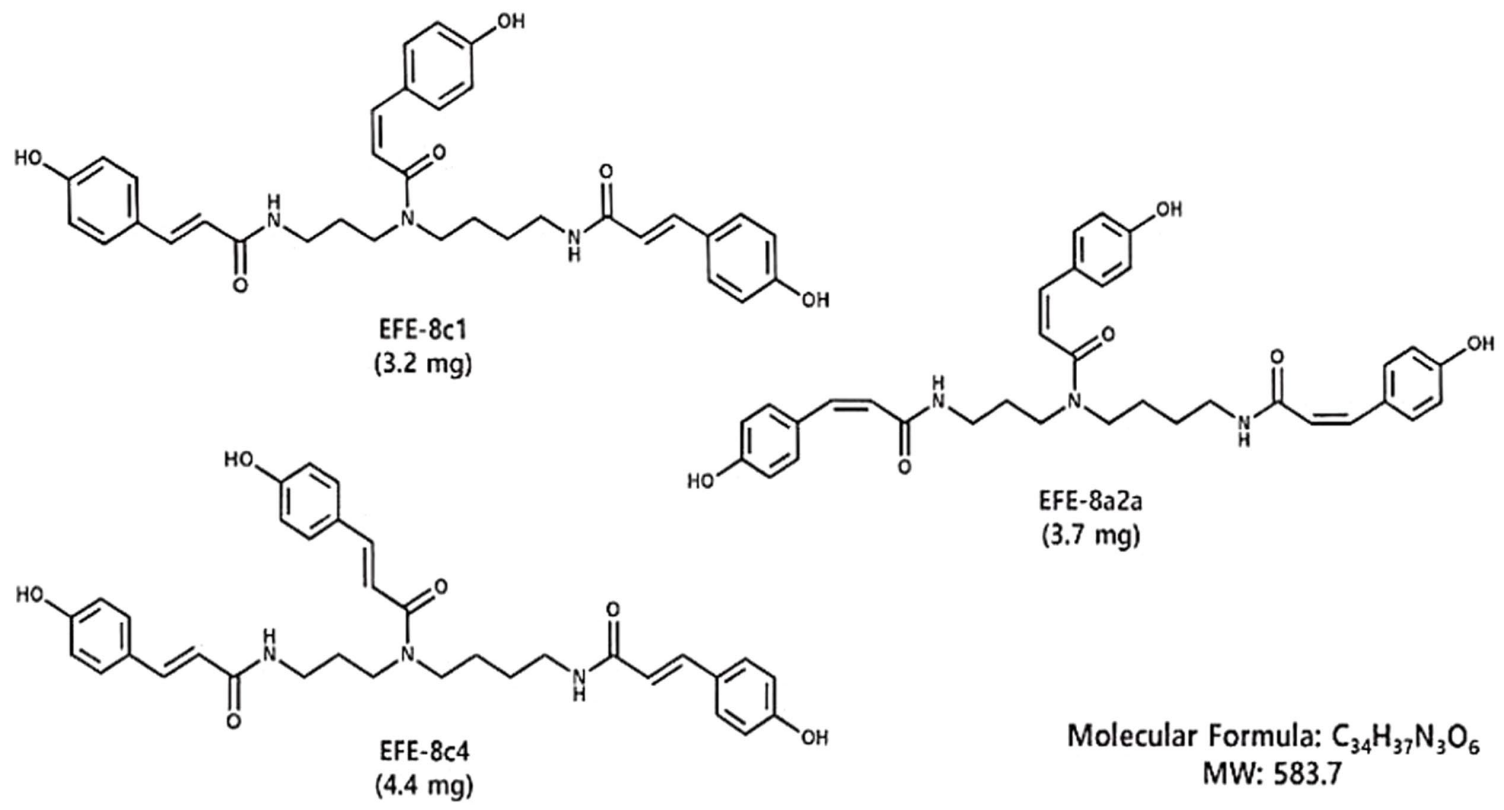
Chemical structures of the three polyamine compounds used in this study. The structures of EFE-8c1, EFE-8a2a, and EFE-8c4 are shown.

**Figure 2:**
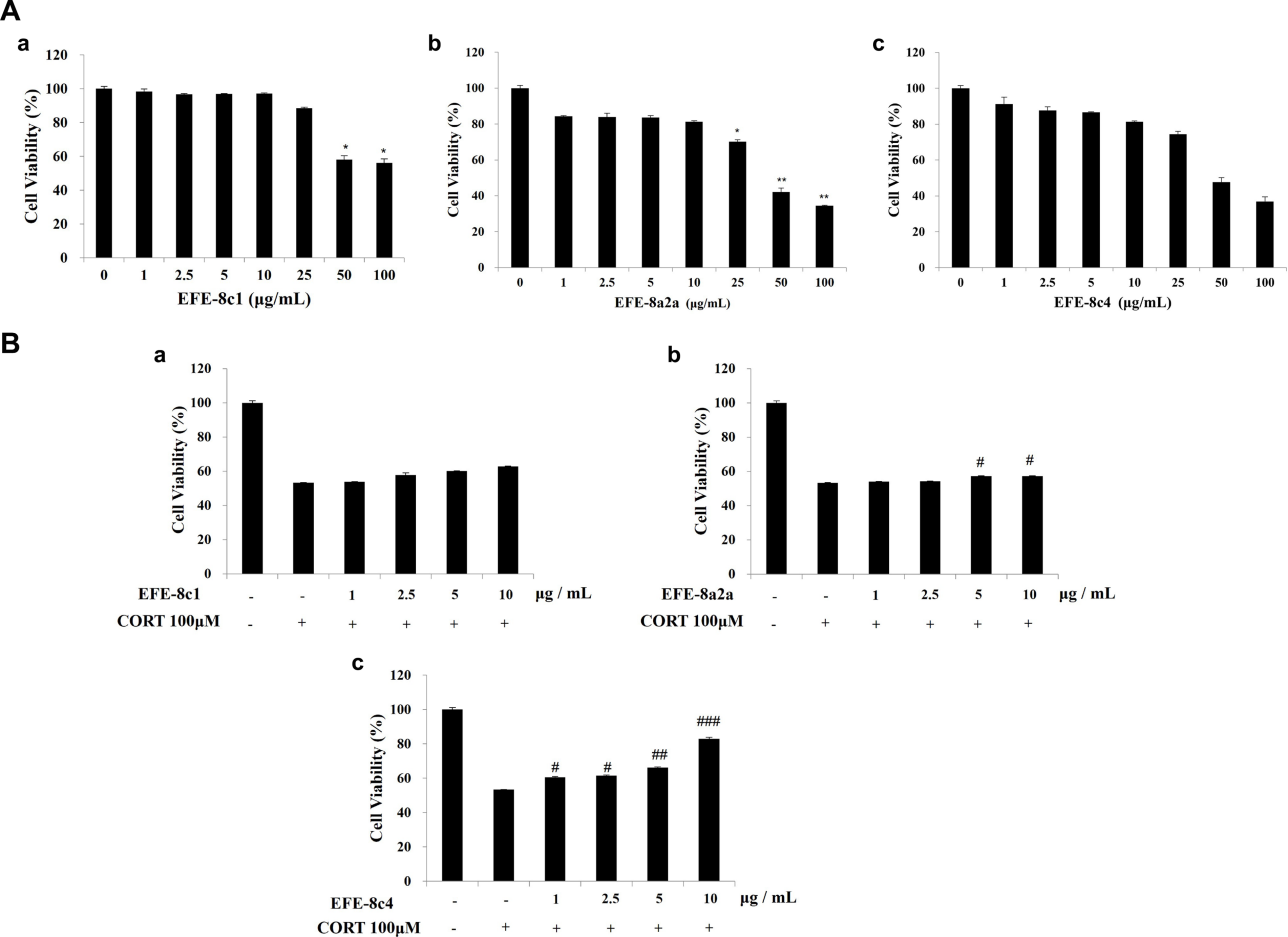
Effects of three single compounds on cell viability and neuroprotective activity in SH-SY5Y cells. **(A)** Cell viability was assessed using the MTS assay. Cytotoxic effects of EFE-8c1 (a), EFE-8a2a (b), and EFE-8c4 (c) at the indicated concentrations. **(B)** Protective effects of the compounds against corticosterone (CORT) induced cytotoxicity in SH-SY5Y cells. Data are presented as mean ± SEM (*n* = 3). Statistical analyses were performed using one-way ANOVA followed by Dunnett’s *post hoc* test. *p < 0.05, **p < 0.01, ***p < 0.001 vs. CORT-treated group.

Three polyamine compounds (EFE-8c1, EFE-8a2a, and EFE-8c4) were isolated from *E. multiflora* fruit and used for subsequent analyses (Figure 1). To establish appropriate experimental conditions, the cytotoxic effects of H₂O_2_ and corticosterone (CORT) were first evaluated in SH-SY5Y cells (Supplementary Figure 1). Both H₂O₂ and CORT induced a dose-dependent reduction in cell viability. Treatment with H₂O₂ resulted in a concentration-dependent decrease in cell viability, with approximately 60% viability observed at 100 μM and approximately 50% at 250 μM, while higher concentrations caused more severe cytotoxicity. Similarly, CORT treatment induced a dose-dependent reduction in cell viability, with approximately 55–60% viability observed at 100 μM. Based on these findings, H₂O₂ (250 μM) and CORT (100 μM) were selected for subsequent experiments, as these concentrations induced moderate but reproducible cytotoxicity without excessive cell death. The cytotoxicity of the three polyamine compounds (EFE-8c1, EFE-8a2a, and EFE-8c4) was then assessed in differentiated SH-SY5Y cells using the MTS assay (Figure 2A). Treatment with these compounds at concentrations ranging from 1 to 10 μg/mL for 24 h did not significantly affect cell viability compared to the vehicle control (DMSO ≤ 0.1%), with cell viability remaining above approximately 80–90%. These results indicate that all three compounds are non-cytotoxic within the tested concentration range, and concentrations up to 10 μg/mL were therefore used in subsequent neuroprotection experiments.

### 3.2 Neuroprotective effects against CORT-induced neurotoxicity

SH-SY5Y cells were exposed to corticosterone (CORT, 100 μM) for 24 h to induce glucocorticoid-mediated neuronal stress. Pretreatment with EFE-8c1 resulted in a moderate, concentration-dependent recovery of cell viability, with significant effects observed at 2.5–10 μg/mL (#*p* < 0.05, ##*p* < 0.01 vs. CORT), reaching approximately 60% at 10 μg/mL. In contrast, EFE-8a2a exhibited minimal protective effects against CORT-induced cytotoxicity. Although slight increases in cell viability were observed at higher concentrations (5–10 μg/mL, #*p* < 0.05 vs. CORT), no clear concentration-dependent trend was evident. Notably, EFE-8c4 demonstrated the most pronounced protective effect among the tested compounds, significantly restoring cell viability in a concentration-dependent manner. Cell viability increased from approximately 53% to approximately 70% at 5–10 μg/mL (##*p* < 0.01, ###*p* < 0.001 vs. CORT) (Figure 2B). Based on its superior protective efficacy, EFE-8c4 was selected for subsequent mechanistic analyses.

### 3.3 EFE-8c4 modulates apoptotic protein expression in H₂O₂-treated cells

**Figure 3:**
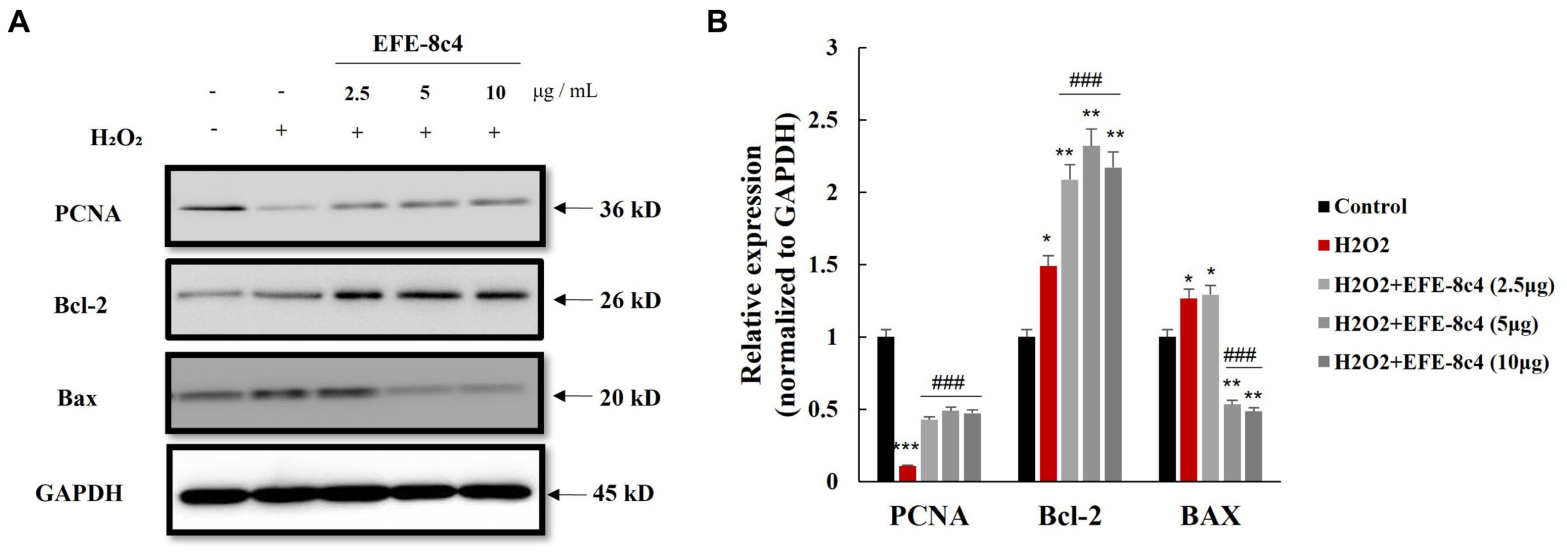
Effects of EFE-8c4 on apoptosis-related protein expression in H₂O₂-treated SH-SY5Y cells. **(A)** Representative immunoblot images showing the expression levels of PCNA, Bcl-2, and Bax in SH-SY5Y cells treated with H₂O₂ in the presence or absence of EFE-8c4 at the indicated concentrations. GAPDH was used as a loading control. **(B)** Quantitative densitometric analysis of PCNA, Bcl-2, and Bax protein levels. Protein expression levels were normalized to GAPDH and expressed as fold change relative to the control group. Data are presented as mean ± SEM (n = 3). Statistical analyses were performed using one-way ANOVA followed by Dunnett’s post hoc test. Significant group effects were observed for PCNA (*F*(4,10) = 4678.0, *p* < 0.001), Bcl-2 (*F*(4,10) = 474.2, *p* < 0.001), and Bax (*F*(4,10) = 634.8, *p* < 0.001). *p < 0.05, **p < 0.01, ***p < 0.001 vs. control group; ###*p* < 0.001 vs. H₂O₂-treated group.

**Figure 4:**
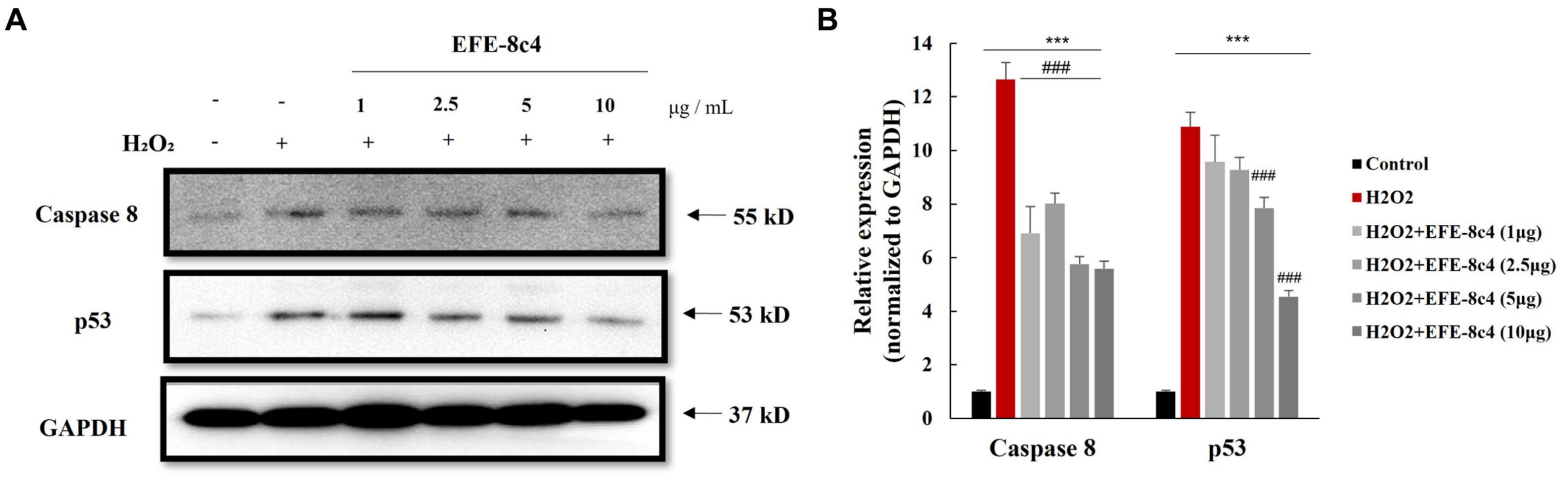
Effects of EFE-8c4 on apoptosis-related protein expression in H₂O₂-treated SH-SY5Y cells. **(A)** Representative immunoblot images showing the expression levels of caspase-8 and p53 in SH-SY5Y cells treated with H₂O₂ in the presence or absence of EFE-8c4 at the indicated concentrations. GAPDH was used as a loading control. **(B)** Quantitative densitometric analysis of caspase-8 and p53 protein levels. Protein expression levels were normalized to GAPDH and expressed as fold change relative to the control group. Data are presented as mean ± SEM (n = 3). Statistical analyses were performed using one-way ANOVA followed by Dunnett’s post hoc test. Significant group effects were observed for caspase-8 (*F*(5,12) = 826.7, *p* < 0.001) and p53 (*F*(5,12) = 376.0, *p* < 0.001). *p < 0.05, **p < 0.01, ***p < 0.001 vs. control group; ###*p* < 0.001 vs. H₂O₂-treated group.

To investigate the molecular mechanisms of EFE-8c4-mediated neuroprotection, Western blot analysis was performed in SH-SY5Y cells. In H₂O₂-treated cells, exposure to H₂O₂ (250 μM, 24 h) significantly decreased the expression of the anti-apoptotic protein Bcl-2 and increased the expression of the pro-apoptotic protein Bax, resulting in a markedly reduced Bcl-2/Bax ratio, consistent with activation of the intrinsic apoptotic pathway (Youle and Strasser, 2008; Hardwick and Soane, 2013). In addition, H₂O₂ treatment reduced PCNA expression, indicating impaired cell survival and proliferative capacity. Representative immunoblot images (Figure 3A) and quantitative densitometric analysis (Figure 3B) demonstrated that EFE-8c4 (2.5–10 μg/mL) dose-dependently restored Bcl-2 expression and suppressed Bax upregulation while significantly increasing PCNA expression. Significant group effects were observed for PCNA (*F*(4,10) = 4678.0, *p* < 0.001), Bcl-2 (F(4,10) = 474.2, *p* < 0.001), and Bax (F(4,10) = 634.8, *p* < 0.001). *Post hoc* analysis demonstrated significant differences compared with the H₂O₂-treated group, as indicated in Figure 3B. To further investigate the downstream apoptotic signaling pathways, the expression levels of caspase-8 and p53 were analyzed by Western blotting. Representative immunoblot images are shown in (Figure 4A), and quantitative densitometric analysis (Figure 4B) confirmed that EFE-8c4 treatment (1–10 μg/mL) significantly attenuated H₂O₂-induced upregulation of caspase-8 and p53 in a dose-dependent manner. Significant group effects were observed for caspase-8 (*F*(5,12) = 826.7, *p* < 0.001) and p53 (F(5,12) = 376.0, *p* < 0.001). Post hoc analysis demonstrated significant differences compared with the H₂O₂-treated group, as indicated in Figure 4B. These findings suggest that EFE-8c4 suppresses oxidative stress-induced apoptotic signaling by inhibiting caspase-8 and p53 activation, thereby contributing to its neuroprotective effects (Youle and Strasser, 2008; Hardwick and Soane, 2013; Salvesen and Dixit, 1999; Vousden and Lane, 2007).

### 3.4 EFE-8c4 restores PCNA expression in H₂O₂ treated cells

**Figure 5:**
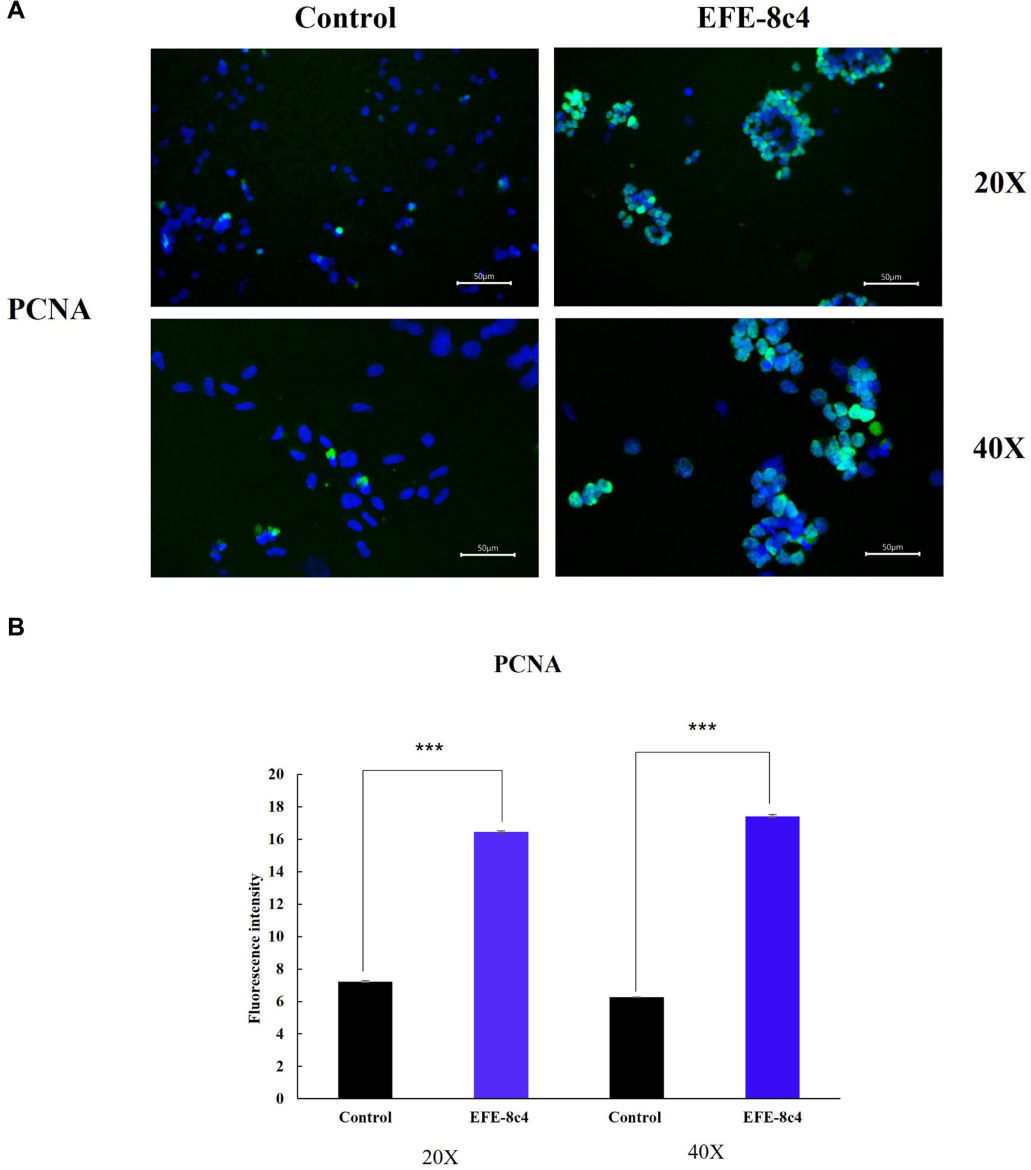
Effects of EFE-8c4 on PCNA expression in SH-SY5Y cells. **(A)** Representative immunofluorescence images of PCNA (green) and nuclei stained with DAPI (blue) in control and EFE-8c4-treated cells (10 μg/mL) for 24 h. Images were captured at 20 × and 40 × magnifications. Scale bars: 50 μm. **(B)** Quantification of PCNA fluorescence intensity. Data are presented as mean ± SEM (n = 3). Statistical analyses were performed using one-way ANOVA followed by Dunnett’s post hoc test. *** *p* < 0.001 vs. control.

PCNA is a well-established marker of cell proliferation and DNA repair activity (Kang and Myung, 2024; Strzalka and Ziemienowicz, 2011). Exposure to H₂O₂ (250 μM, 24 h) significantly reduced PCNA expression in SH-SY5Y cells, indicating impaired cell survival and proliferative capacity under oxidative stress conditions. Western blot analysis demonstrated that treatment with EFE-8c4 (2.5–10 μg/mL) dose-dependently restored PCNA expression toward control levels (###*p* < 0.001 vs. H₂O₂), suggesting that EFE-8c4 mitigates oxidative stress-induced suppression of cell survival pathways. To further validate these findings, immunocytochemical analysis was performed. Representative fluorescence images (Figure 5A) and quantitative analysis (Figure 5B) showed that EFE-8c4 treatment (10 μg/mL, 24 h) significantly increased nuclear PCNA immunofluorescence intensity compared with control cells (p < 0.001). Consistent results were also observed in high-magnification images (Supplementary Figure 2), further supporting the promotive effect of EFE-8c4 on proliferative activity. Collectively, these results indicate that EFE-8c4 restores PCNA expression and enhances cell survival capacity under oxidative stress conditions, potentially contributing to its neuroprotective effects (Kang and Myung, 2024; Strzalka and Ziemienowicz, 2011).

### 3.5 EFE-8c4 partially reduces CORT-induced apoptosis by flow cytometry

**Figure 6:**
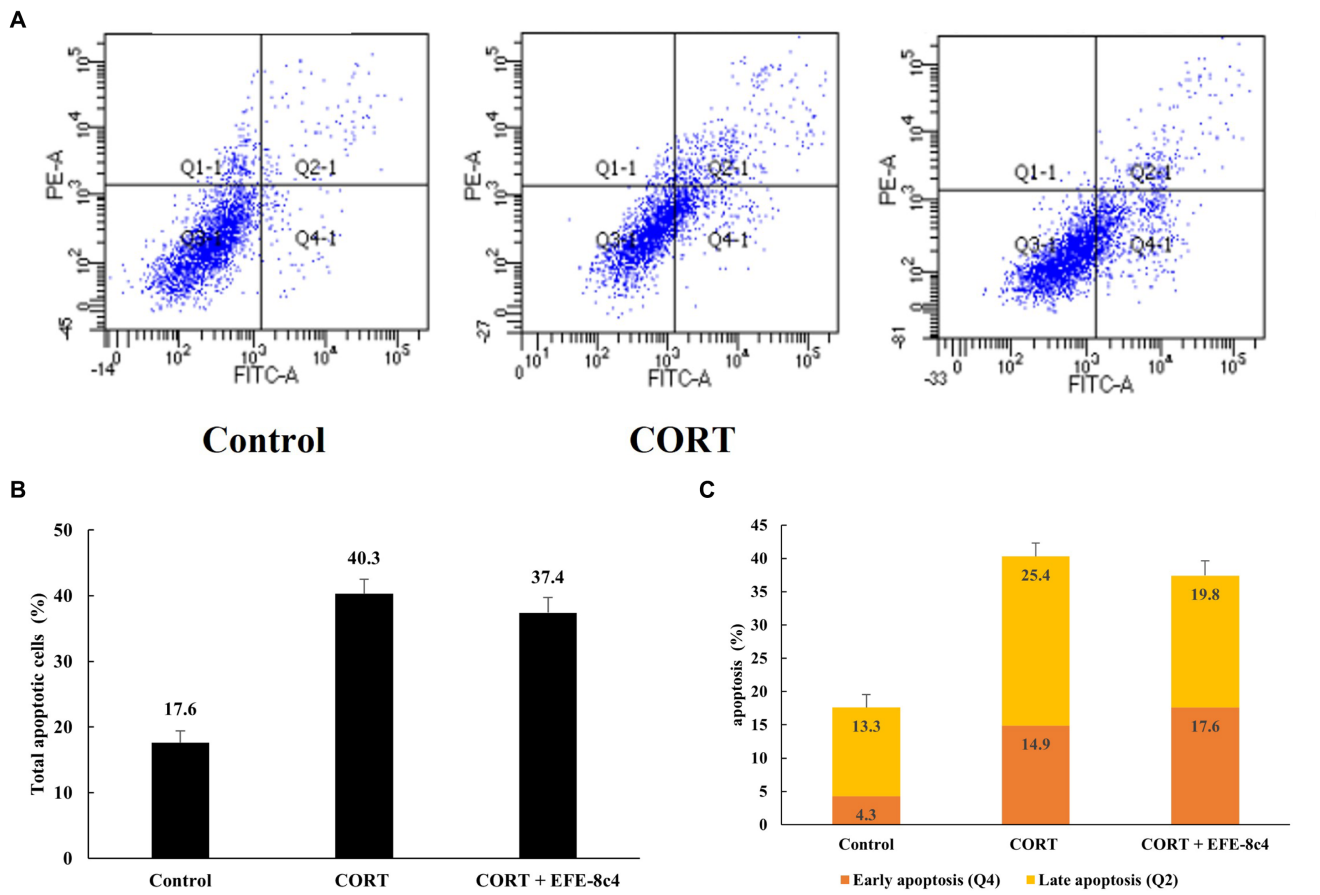
Effects of EFE-8c4 on corticosterone (CORT)-induced apoptosis in SH-SY5Y cells. **(A)** Representative Annexin V-FITC/propidium iodide (PI) flow cytometry dot plots showing apoptotic cell populations in Control, CORT-treated, and CORT + EFE-8c4-treated groups. Quadrants represent necrotic (Q1), late apoptotic (Q2; Annexin V^+^/PI^+^), viable (Q3; Annexin V^−^/PI^−^), and early apoptotic (Q4; Annexin V^+^/PI^−^) cell populations. **(B)** Quantification of total apoptotic cells, calculated as the sum of early and late apoptotic populations (Q4 + Q2). **(C)** Quantitative analysis of early (Q4) and late (Q2) apoptotic cell populations. Data are presented as mean ± SEM (*n* = 3). No statistically significant difference was observed between the CORT and CORT + EFE-8c4 groups.

Flow cytometric analysis using Annexin V-FITC/PI staining was performed to quantify apoptotic cell populations (Figure 6). Representative dot plots (Figure 6A) show the distribution of viable, early apoptotic (Q4), late apoptotic (Q2), and necrotic cells. CORT treatment (200 μM, 24 h) markedly increased the proportion of total apoptotic cells (early + late apoptosis; Q2 + Q4) from approximately 17.6% in control cells to 40.3% (Figure 6B). Treatment with EFE-8c4 (10 μg/mL) modestly reduced total apoptotic cell populations to approximately 37.4%. Further analysis of apoptotic subpopulations (Figure 6C) revealed that CORT treatment significantly increased both early apoptotic (Q4) and late apoptotic (Q2) cell fractions. EFE-8c4 treatment resulted in a reduction in late apoptotic cells, while early apoptotic cells were slightly increased, suggesting a partial attenuation of apoptosis progression rather than complete inhibition. These findings indicate that EFE-8c4 exerts a moderate anti-apoptotic effect under CORT-induced stress conditions. The observed changes are consistent with the anti-apoptotic protein regulation identified in Western blot analyses, supporting the involvement of apoptosis-related signaling pathways, including Bcl-2/Bax and caspase-associated mechanisms (Youle and Strasser, 2008; Hardwick and Soane, 2013; Salvesen and Dixit, 1999).

### 3.6 EFE-8c4 promotes neuronal differentiation marker βIII-tubulin expression

**Figure 7:**
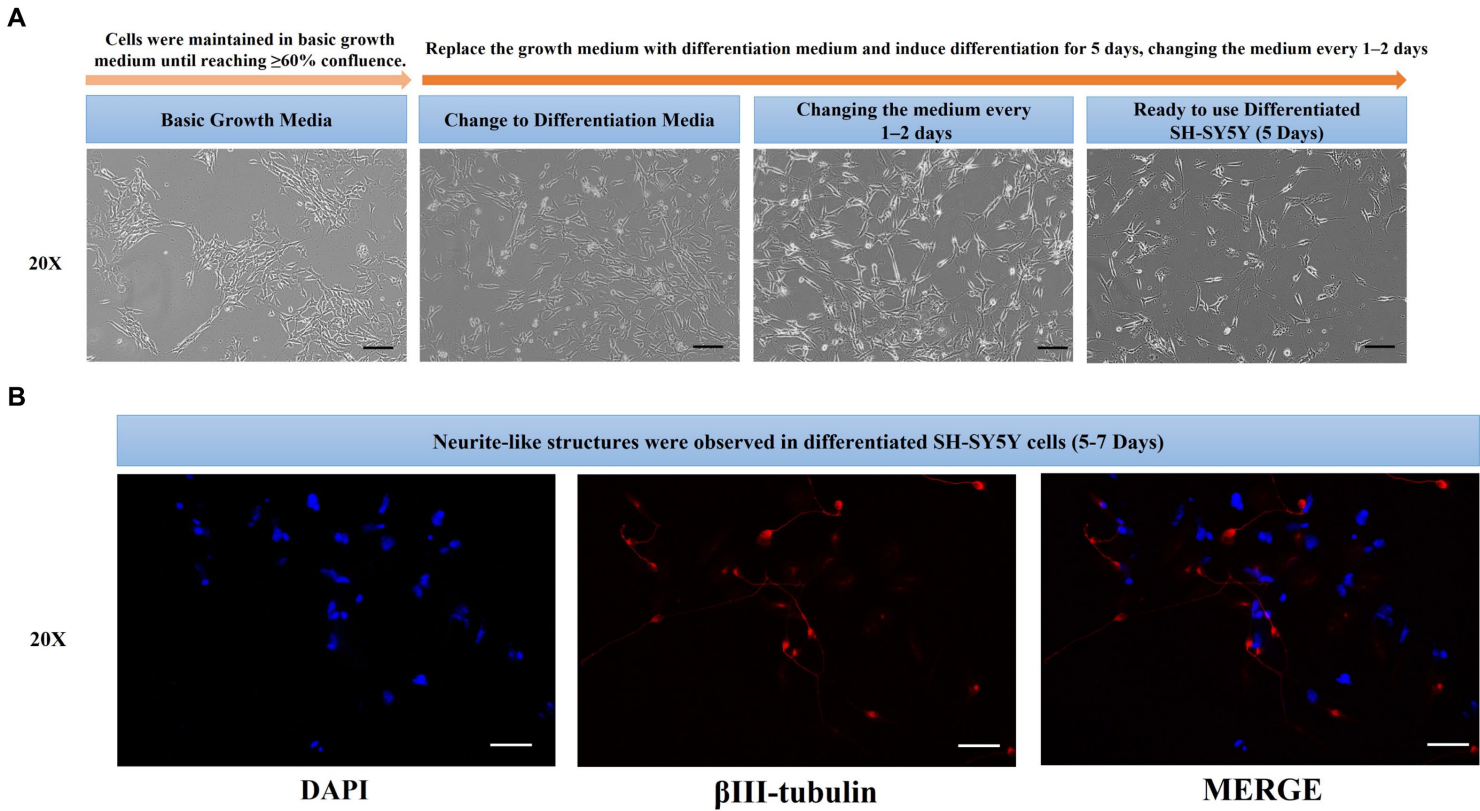
Differentiation of SH-SY5Y cells into neuron-like cells. **(A)** Representative phase-contrast images showing morphological changes during differentiation. Cells were maintained in basic growth medium until approximately 60% confluence, followed by replacement with differentiation medium. Differentiation was induced for 5–7 days, with medium changes every 1–2 days. **(B)** Representative immunofluorescence images of differentiated SH-SY5Y cells showing nuclei stained with DAPI (blue) and the neuronal marker βIII-tubulin (red). The merged images demonstrate neurite-like structures. Images were captured at 20× magnification. Scale bars: 50 μm.

**Figure 8:**
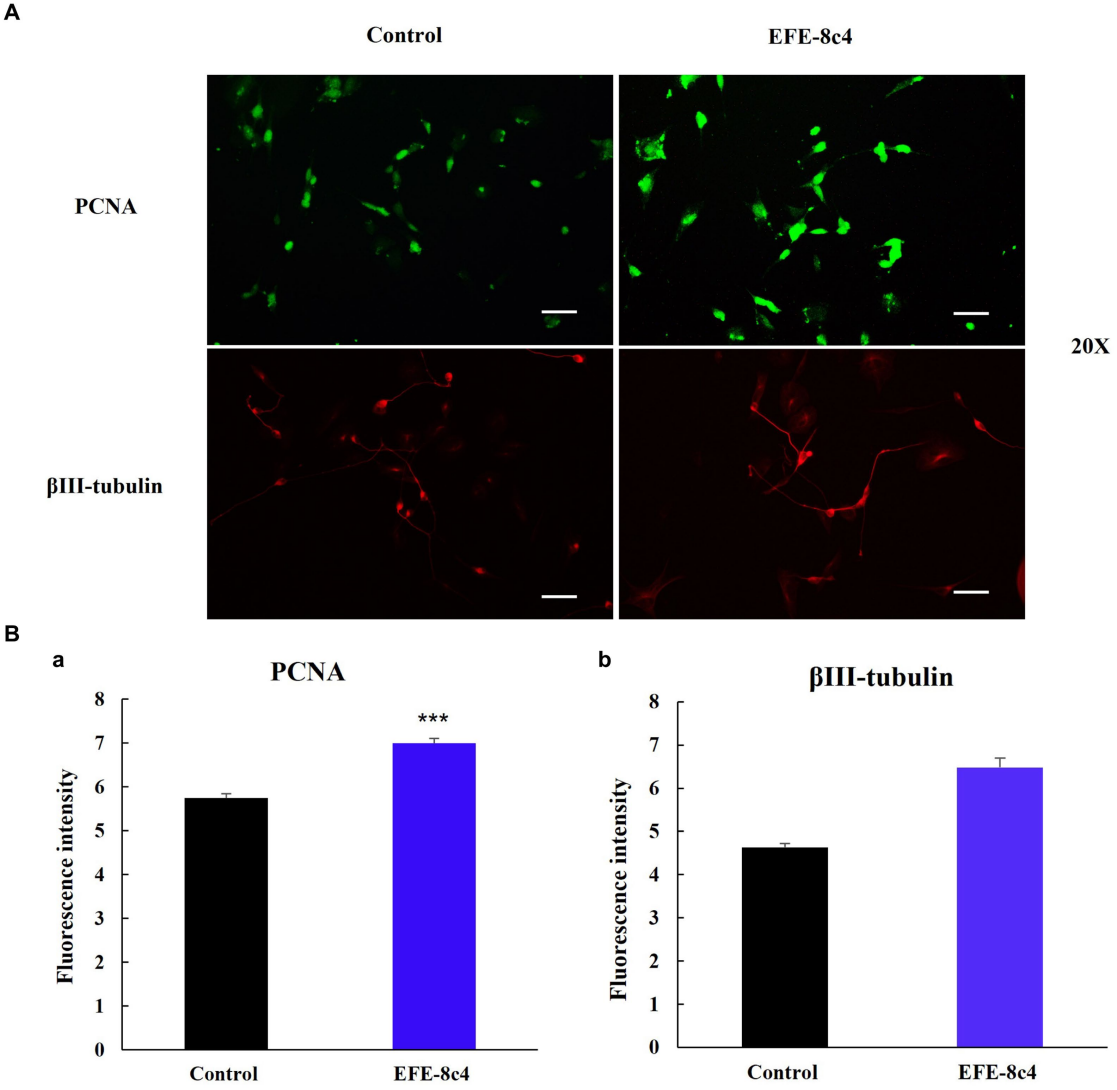
Effects of EFE-8c4 on PCNA and βIII-tubulin expression in differentiated SH-SY5Y cells. **(A)** Representative immunofluorescence images of PCNA (green) and βIII-tubulin (red) in control and EFE-8c4-treated differentiated SH-SY5Y cells (10 μg/mL, 24 h). Nuclei were stained with DAPI (blue). **(B)** Quantification of fluorescence intensity of **(a)** PCNA and **(b)** βIII-tubulin. Data are presented as mean ± SEM (n = 3). Statistical analyses were performed using one-way ANOVA followed by Dunnett’s post hoc test. ***p < 0.001 vs. control. Images were acquired at 20× magnification. Scale bars: 50 μm.

As Neuronal differentiation of SH-SY5Y cells was first confirmed by morphological changes and increased βIII-tubulin expression following retinoic acid (RA) treatment (Figure 7). To further evaluate the effect of EFE-8c4 on neuronal characteristics, immunocytochemical analysis was performed. As shown in Figure 8 treatment with EFE-8c4 (10 μg/mL, 24 h) significantly increased βIII-tubulin immunofluorescence intensity in differentiated SH-SY5Y cells compared to control (*p* < 0.001). βIII-tubulin is a neuron-specific cytoskeletal protein widely used as a marker of neuronal differentiation and structural integrity. The observed increase in βIII-tubulin expression suggests that EFE-8c4 enhances neuronal phenotype stability and supports neuronal structural maintenance under experimental conditions. These findings indicate that, in addition to its anti-apoptotic and cytoprotective effects, EFE-8c4 may contribute to the preservation of neuronal characteristics in differentiated neuronal cells.

### 3.7 EFE-8c4 attenuates H₂O₂ induced reactive oxygen species production

ROS detection using the DCF-DA assay revealed that H₂O₂ treatment (250 μM, 24 h) markedly increased intracellular ROS levels in differentiated SH-SY5Y cells, as evidenced by intense DCF fluorescence (Supplementary Figure 3). EFE-8c4 (10 μg/mL) dose-dependently and markedly reduced H₂O₂-induced DCF fluorescence, indicating effective ROS scavenging activity. These results demonstrate that EFE-8c4 possesses significant antioxidant activity in neuronal cells, consistent with its neuroprotective effects against H₂O₂ induced oxidative stress.

## 4 Discussion

The present study demonstrates that EFE-8c4, a novel polyamine compound isolated from *Elaeagnus multiflora* Thunb. fruit, exhibits potent and multifaceted neuroprotective effects in differentiated SH-SY5Y cells. EFE-8c4 protected against both CORT- and H₂O₂-induced neurotoxicity through multiple complementary mechanisms, including modulation of apoptotic pathways, promotion of cell proliferation, attenuation of calcium dysregulation, reduction of oxidative stress, and enhancement of neuronal differentiation. These findings provide the first evidence for the neuroprotective potential of polyamine compounds from *E. multiflora* and support their further development as therapeutic agents for neurological disorders.

### 4.1 Neuroprotection against CORT-induced neurotoxicity

EFE-8c4 significantly protected differentiated SH-SY5Y cells against CORT-induced cell death, restoring viability from ~50% to ~70% at 5–10 μg/mL. CORT-induced neurotoxicity is a well-established model of glucocorticoid-mediated neuronal damage relevant to stress-related neurological disorders, including depression and post-traumatic stress disorder (Joëls et al., 2018; De Kloet et al., 2005). The mechanism of CORT-induced neurotoxicity involves multiple pathways, including mitochondrial dysfunction, calcium dysregulation, and activation of apoptotic cascades. The ability of EFE-8c4 to counteract these effects suggests that it may target one or more of these pathways, potentially through its polyamine scaffold, which is known to modulate ion channels, receptor activity, and intracellular signaling (Pegg, 2016; Igarashi and Kashiwagi, 2010).

### 4.2 Anti-apoptotic mechanisms

A key finding of this study is that EFE-8c4 reverses H₂O₂-induced apoptotic signaling by restoring the Bcl-2/Bax balance, suppressing caspase-8 activation, and reducing p53 upregulation. The Bcl-2 family of proteins is central to the regulation of the intrinsic apoptotic pathway (Youle and Strasser, 2008; Hardwick and Soane, 2013). Oxidative stress-induced mitochondrial dysfunction is a major upstream trigger of apoptosis (Wang, 2001), and reactive oxygen species (ROS) play a critical role in activating apoptotic signaling cascades (Simon et al., 2000; Circu and Aw, 2010). Recent studies have further highlighted the involvement of polyamine-associated signaling pathways in neuronal apoptosis and neurodegenerative progression (Yan et al., 2026). The restoration of Bcl-2 expression and suppression of Bax by EFE-8c4 suggests that it stabilizes mitochondrial function and prevents the initiation of the intrinsic apoptotic cascade.

### 4.3 Promotion of cell proliferation and DNA repair

EFE-8c4 significantly restored PCNA expression in H₂O₂-treated cells and increased PCNA immunofluorescence in untreated cells. PCNA is an essential component of the DNA replication machinery and participates in multiple DNA repair pathways (Kang and Myung, 2024; Strzalka and Ziemienowicz, 2011). The upregulation of PCNA by EFE-8c4 suggests that it promotes DNA repair and cell cycle progression in oxidatively stressed neurons, thereby enhancing neuronal survival and recovery. This effect is consistent with the known roles of polyamines in stimulating cell proliferation and DNA synthesis (Pegg, 2016; Igarashi and Kashiwagi, 2010).

### 4.4 Antioxidant effects and ROS scavenging

EFE-8c4 markedly reduced H₂O₂-induced ROS production in differentiated SH-SY5Y cells. Oxidative stress, characterized by excessive ROS generation and depletion of antioxidant defenses, is a central mechanism of neuronal damage in neurodegenerative diseases, depression, and acute neurological injuries (Olufunmilayo et al., 2023; Angelova and Abramov, 2016; Simon et al., 2000; Circu and Aw, 2010). Recent reviews further support oxidative stress as a major driver of neurodegenerative progression and antioxidant-based neuroprotection (Houldsworth, 2024). The antioxidant effects of EFE-8c4 may be attributable to its polyamine scaffold, which can directly scavenge free radicals and indirectly regulate endogenous antioxidant systems, including glutathione metabolism (Halliwell, 2001; Birben et al., 2012; Dringen, 2000). Recent evidence using SH-SY5Y neuronal models has demonstrated that natural product-derived compounds attenuate oxidative stress-induced neuronal damage through antioxidant and cytoprotective mechanisms (Jongwachirachai et al., 2024; Alvariño et al., 2024). These findings support the broad neuroprotective potential of EFE-8c4 against ROS-mediated neuronal injury.

### 4.5 Promotion of neuronal differentiation

EFE-8c4 significantly increased βIII-tubulin expression in differentiated SH-SY5Y cells. βIII-tubulin is a neuron-specific tubulin isoform that plays essential roles in axonal growth, neuronal migration, and maintenance of neuronal morphology (Radwitz et al., 2022). Enhanced βIII-tubulin expression indicates that EFE-8c4 promotes neuronal differentiation and may support axonal integrity and neurite outgrowth. These effects are consistent with the known roles of polyamines in promoting neuronal differentiation and synaptic plasticity (Pegg, 2016; Minois et al., 2011).

### 4.6 Relevance to natural product-based neuroprotection

The neuroprotective effects of EFE-8c4 are consistent with a growing body of literature demonstrating the therapeutic potential of plant-derived compounds for neurological disorders (Olufunmilayo et al., 2023; Houldsworth, 2024). Natural products from traditional medicinal plants have yielded numerous neuroprotective compounds, including alkaloids, flavonoids, terpenoids, and polyamines, that target multiple pathways relevant to neurodegeneration (Madeo et al., 2018; Olufunmilayo et al., 2023). Recent investigations have increasingly focused on multifunctional natural compounds capable of simultaneously modulating oxidative stress, apoptosis, and mitochondrial dysfunction in neuronal cells (Güner and Tekin, 2025). The polyamine scaffold of EFE-8c4, combined with its aromatic substituents (C34H37N3O6), may confer both direct antioxidant activity and the ability to modulate receptor and enzyme function, as observed in this study. The traditional use of *E. multiflora* in East Asian medicine for anti-inflammatory and immunomodulatory purposes (Sapolsky, 2000; McEwen et al., 2016) is consistent with the neuroprotective activities observed here.

### 4.7 Therapeutic implications and future directions

The multifaceted neuroprotective effects of EFE-8c4 suggest potential therapeutic applications in several neurological disorders. First, neurodegenerative diseases such as Alzheimer’s disease and Parkinson’s disease are characterized by oxidative stress, apoptosis, and neuronal loss (GBD 2021 Nervous System Disorders Collaborators, 2024; Dugger and Dickson, 2017; Bredesen et al., 2006; Lin and Beal, 2006; Barnham et al., 2004). The anti-apoptotic, antioxidant, and pro-proliferative effects of EFE-8c4 may therefore be relevant to these conditions. Second, depression and stress-related disorders involve chronic stress and elevated glucocorticoids as major risk factors, and compounds that protect against glucocorticoid-induced neuronal damage may have therapeutic value (Halliwell, 2006; Joëls et al., 2018). Third, acute neurological injuries such as stroke and traumatic brain injury involve oxidative stress and excitotoxicity, and antioxidant compounds capable of suppressing ROS-mediated apoptosis may provide neuroprotection in these acute settings (Olufunmilayo et al., 2023; Houldsworth, 2024; Jongwachirachai et al., 2024; Alvariño et al., 2024). Future studies should focus on *in vivo* validation using animal models of neurodegeneration, depression, and acute neurological injury to evaluate pharmacokinetics, bioavailability, and safety.

### 4.8 Limitations

This study has several limitations that should be acknowledged. First, the experiments were conducted in a 2D monoculture system using a single cell line (SH-SY5Y), which does not fully recapitulate the complexity of the brain microenvironment. Future studies should employ more complex models, such as cocultures with astrocytes and microglia (Park et al., 2024), ex vivo brain slice cultures. Second, the molecular targets and detailed mechanisms of action of EFE-8c4 remain to be fully elucidated. Third, *in vivo* validation in animal models is needed to confirm the neuroprotective effects observed *in vitro* and to assess pharmacokinetics, bioavailability, and potential side effects. Fourth, the structural characterization of the three polyamine compounds is incomplete, and further studies are needed to determine their exact chemical structures and stereochemistry.

## 5 Conclusion

In conclusion, EFE-8c4, a polyamine compound derived from *Elaeagnus multiflora* Thunb. fruit, protected SH-SY5Y neuronal cells against corticosterone- and hydrogen peroxide-induced cellular stress. EFE-8c4 attenuated oxidative stress-associated damage by reducing ROS accumulation and modulating apoptosis-related signaling, including restoration of the Bcl-2/Bax balance and suppression of caspase-8 and p53 expression. EFE-8c4 also enhanced PCNA and βIII-tubulin expression, suggesting improved cell survival capacity and maintenance of neuronal characteristics. These findings support EFE-8c4 as a promising natural product-derived neuroprotective candidate; however, further studies are required to validate its efficacy, pharmacokinetic properties, and safety *in vivo* models.

## Data Availability

The original contributions presented in the study are included in the article/Supplementary material, further inquiries can be directed to the corresponding author.

## Supplementary material

**SUPPLEMENTARY FIGURE 1**

Cytotoxic concentrations of H₂O₂ and CORT in differentiated SH-SY5Y cells. **(A)** Cell viability following treatment with increasing concentrations of H₂O₂ (100, 250, 500, 1,000 μM) for 24 h. **(B)** Cell viability following treatment with increasing concentrations of CORT (100, 200, 1,000 μM) for 24 h. Cell viability was assessed by MTS assay. Data are presented as mean ± SEM (*n* = 3). Statistical analyses were performed using one-way ANOVA followed by Dunnett’s *post hoc* test. ****p* < 0.001 vs. control.

**SUPPLEMENTARY FIGURE 2**

EFE-8c4 enhances PCNA expression in differentiated SH-SY5Y cells (replicate experiment). **(A)** Representative immunofluorescence images showing PCNA (green) and DAPI (blue) in control and EFE-8c4-treated (20 μg/mL) cells at 40× magnification. Scale bars: 50 μm. **(B)** Quantification of PCNA fluorescence intensity. Data are presented as mean ± SEM (*n* = 3). Statistical analyses were performed using one-way ANOVA followed by Dunnett’s *post hoc* test. ****p* < 0.001 vs. control.

**SUPPLEMENTARY FIGURE 3**

Detection of intracellular reactive oxygen species (ROS) in SH-SY5Y cells. **(A,B)** Representative fluorescence images of ROS levels using DCF-DA (green) and nuclei stained with DAPI (blue) in control, H₂O₂-treated, and H₂O₂ + EFE-8c4-treated cells. Images were captured at 20× **(A)** and 40× **(B)** magnifications. Scale bars: 50 μm. Statistical analyses were performed using one-way ANOVA followed by Dunnett’s *post hoc* test. ****p* < 0.001 vs. control.
