# Supplementary figures and images for: EFE-8c4, a polyamine from Elaeagnus multiflora, protects neuronal cells by regulating oxidative stress and apoptotic pathways
Source: Front Neurosci. 2026 Jul 2;20:1868462. doi: 10.3389/fnins.2026.1868462 (PMC13372788; doi:10.3389/fnins.2026.1868462)

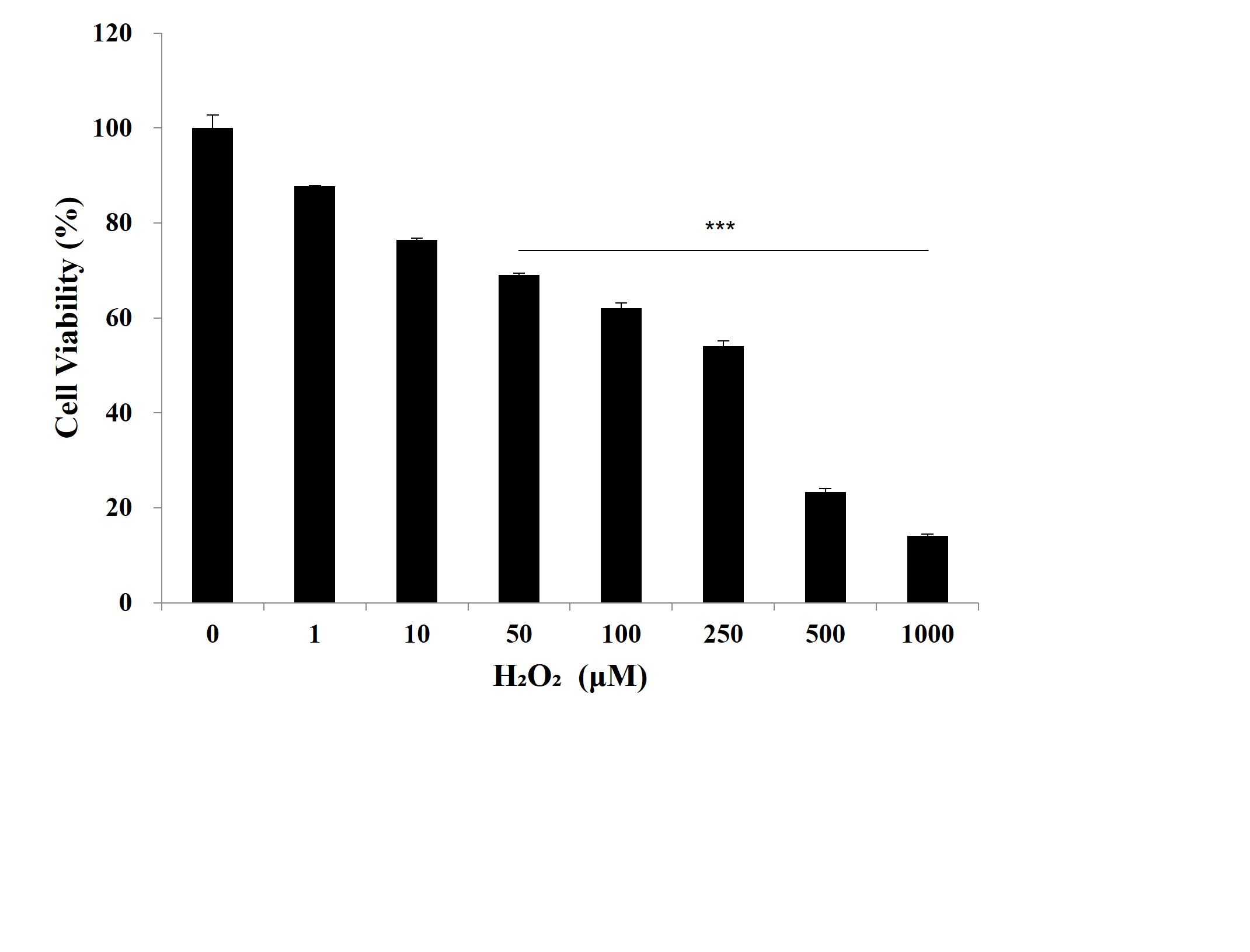

Supplement: Supplementary file 1 [file Supplementary_file_1.zip › Supplementary Figure 1A.jpg]

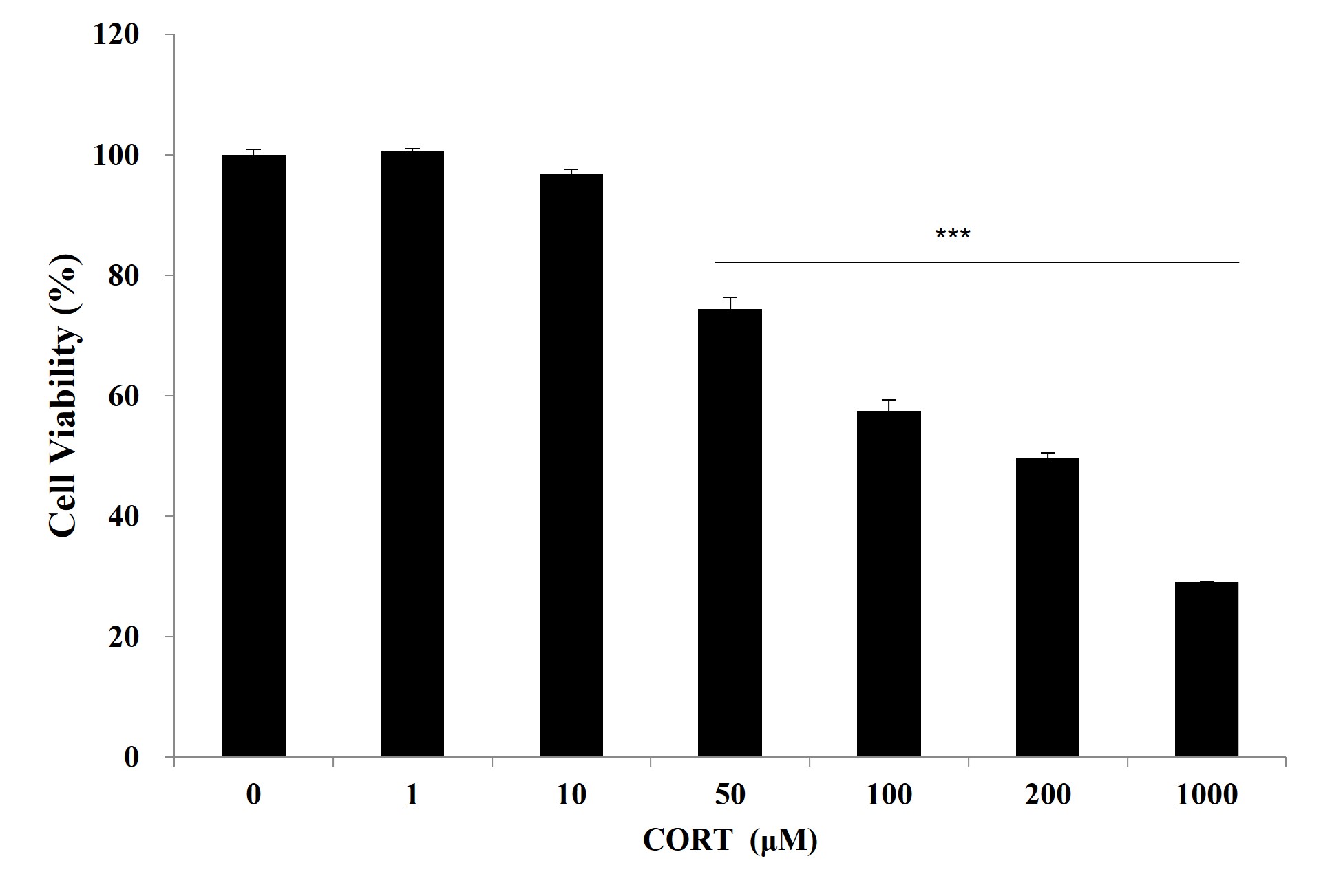

Supplement: Supplementary file 1 [file Supplementary_file_1.zip › Supplementary Figure 1B.jpg]

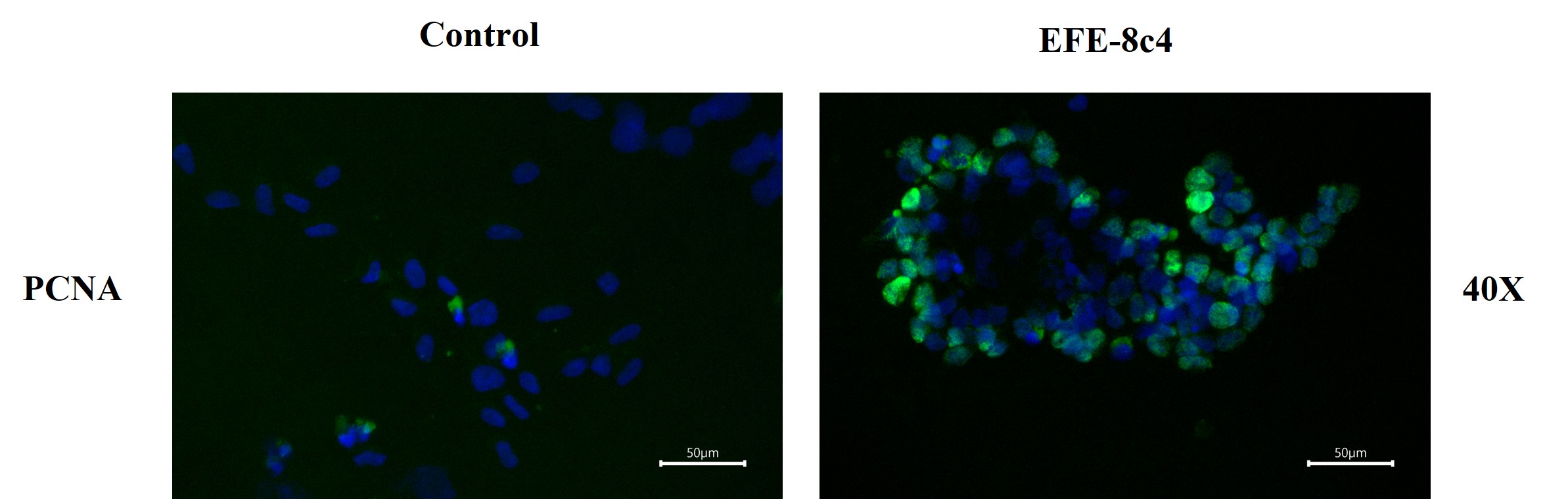

Supplement: Supplementary file 1 [file Supplementary_file_1.zip › Supplementary Figure 2A.jpg]

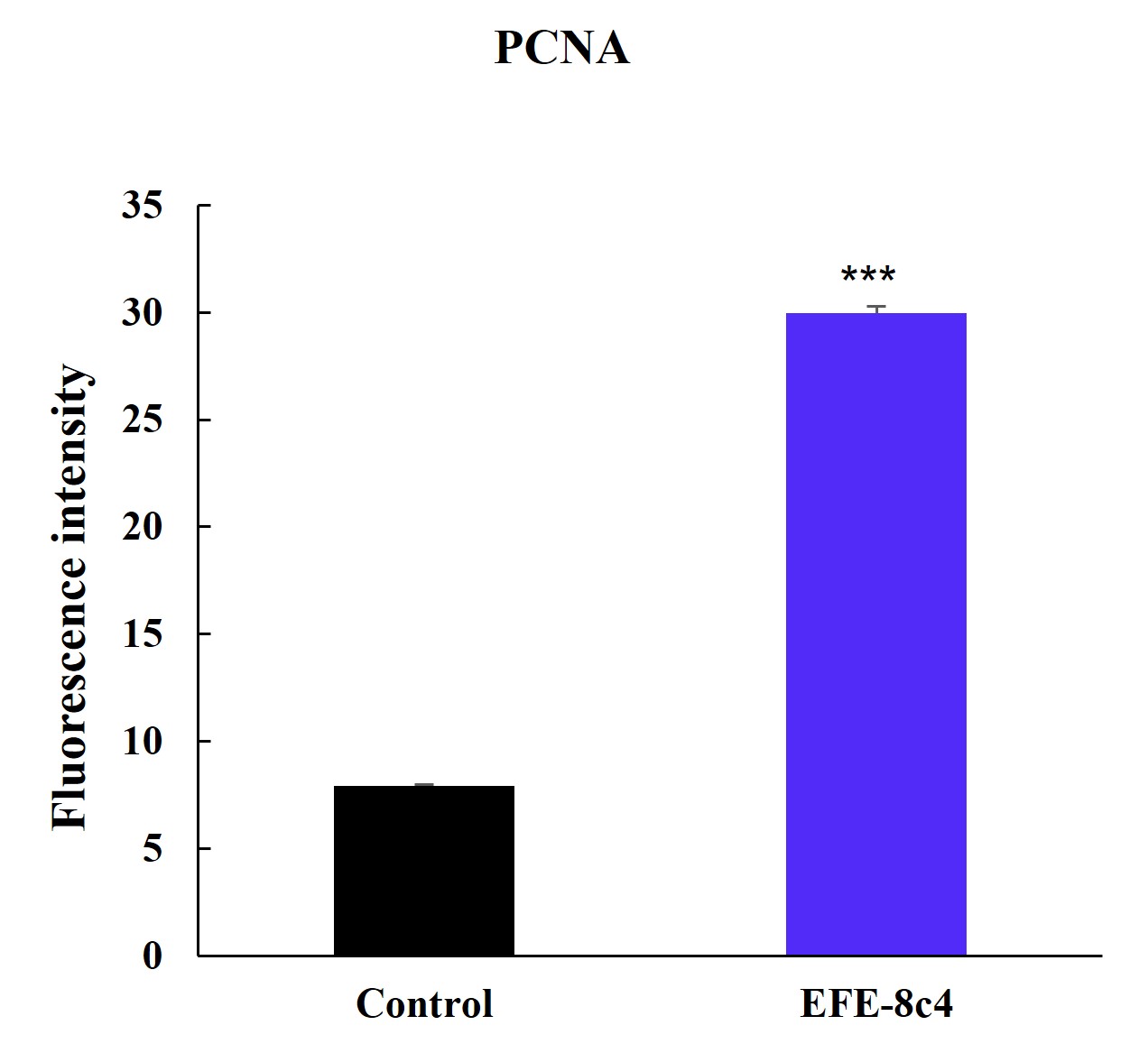

Supplement: Supplementary file 1 [file Supplementary_file_1.zip › Supplementary Figure 2B.jpg]

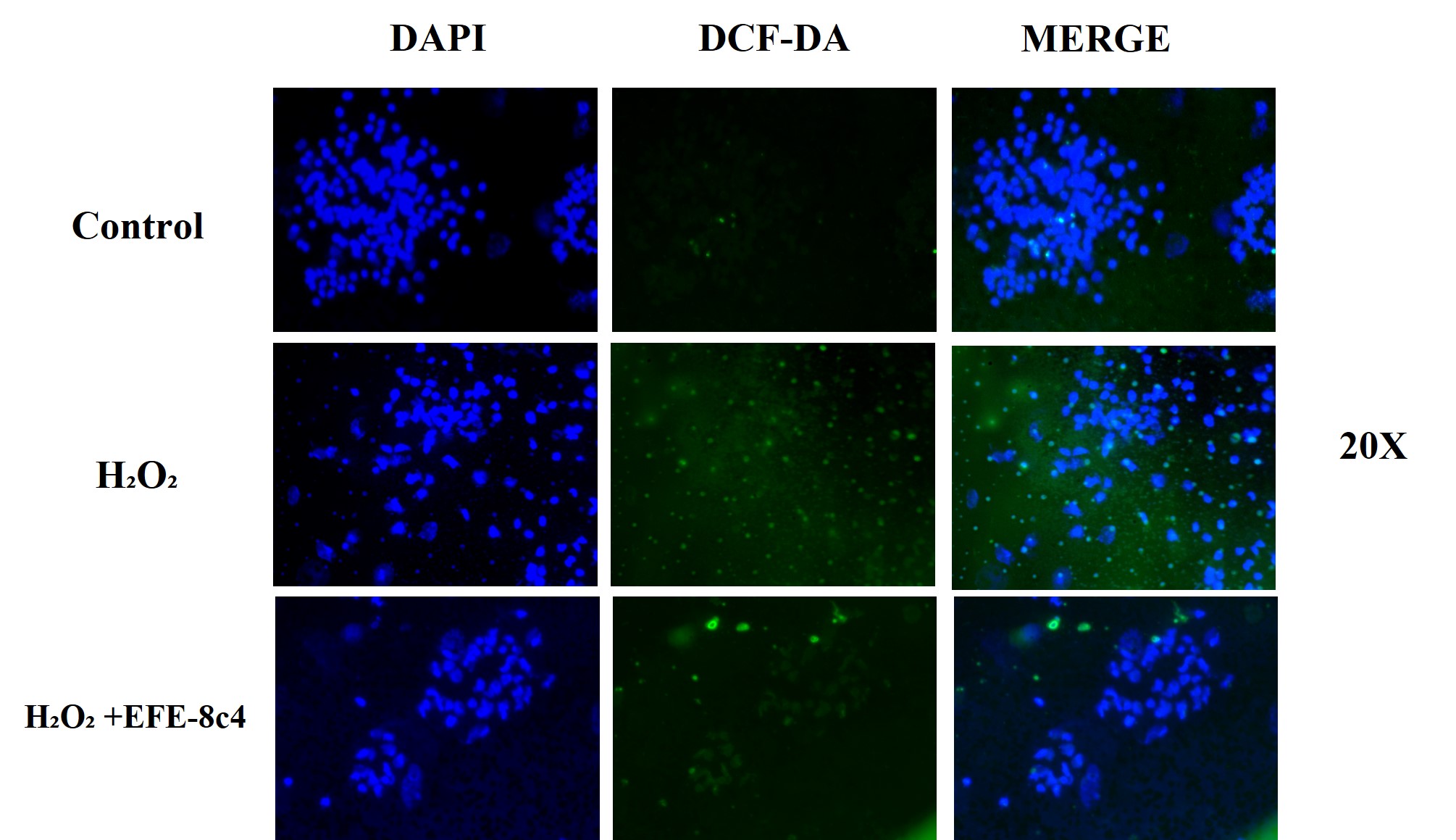

Supplement: Supplementary file 1 [file Supplementary_file_1.zip › Supplementary Figure 3A.jpg]

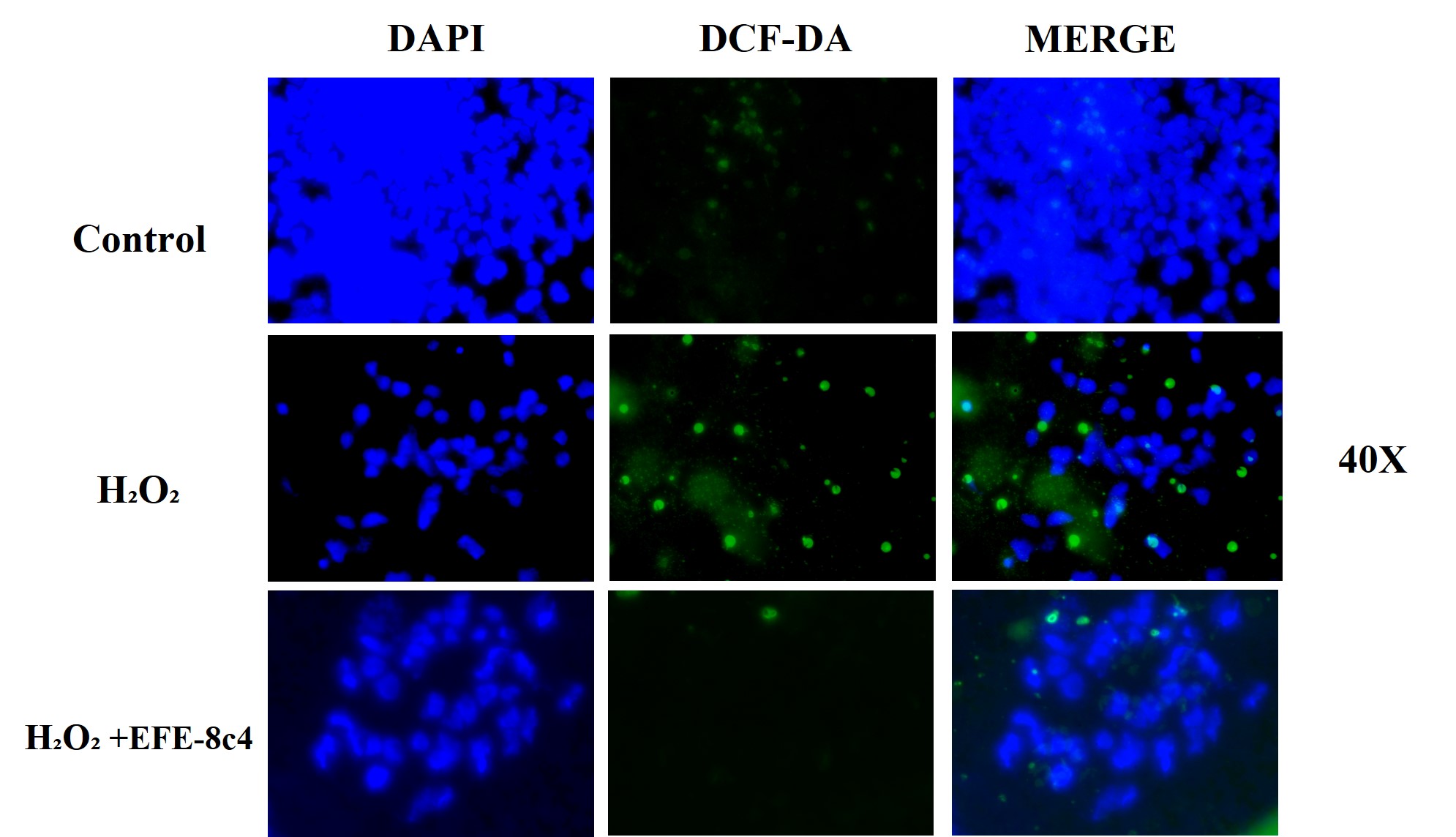

Supplement: Supplementary file 1 [file Supplementary_file_1.zip › Supplementary Figure 3B.jpg]

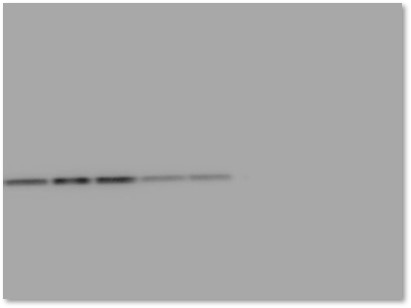

Supplement: Supplementary file 2 [file Supplementary_file_2.ZIP › supplement Figure/Figure 3. WBA BAX Membrane.jpg]

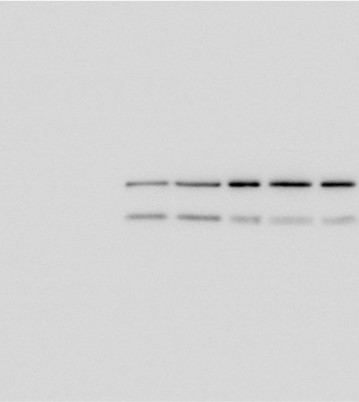

Supplement: Supplementary file 2 [file Supplementary_file_2.ZIP › supplement Figure/Figure 3. WBA Bcl-2 Membrane_upper lane.jpg]

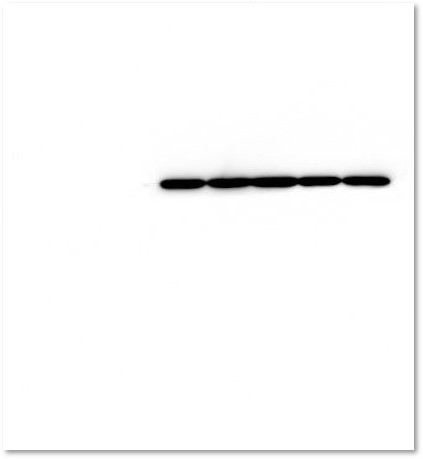

Supplement: Supplementary file 2 [file Supplementary_file_2.ZIP › supplement Figure/Figure 3. WBA GAPDH Membrane.jpg]

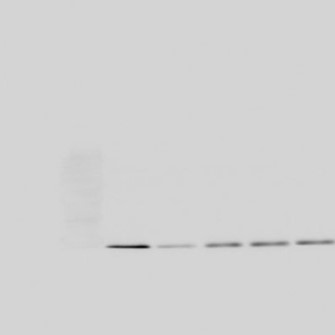

Supplement: Supplementary file 2 [file Supplementary_file_2.ZIP › supplement Figure/Figure 3. WBA PCNA Membrane.jpg]

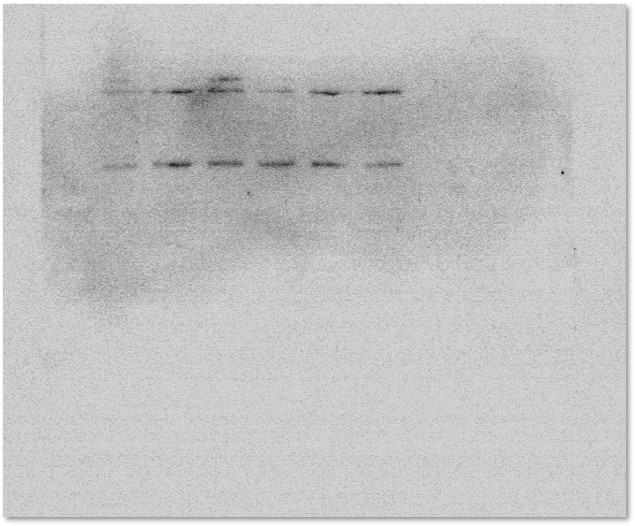

Supplement: Supplementary file 2 [file Supplementary_file_2.ZIP › supplement Figure/Figure 4. WBA Caspase 8 Membrane_under lane.jpg]

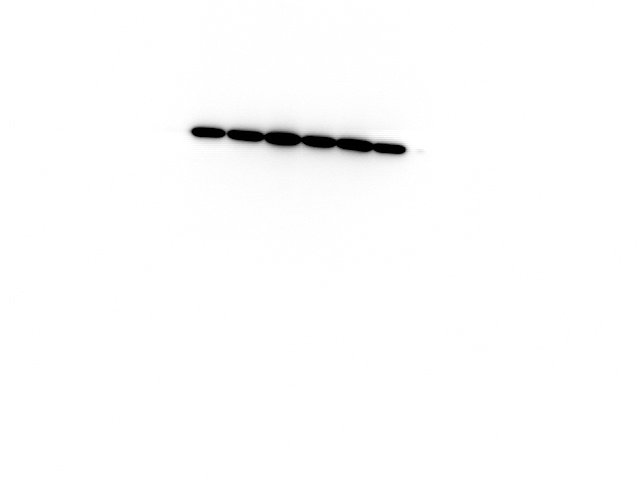

Supplement: Supplementary file 2 [file Supplementary_file_2.ZIP › supplement Figure/Figure 4. WBA GAPDH Membrane.jpg]

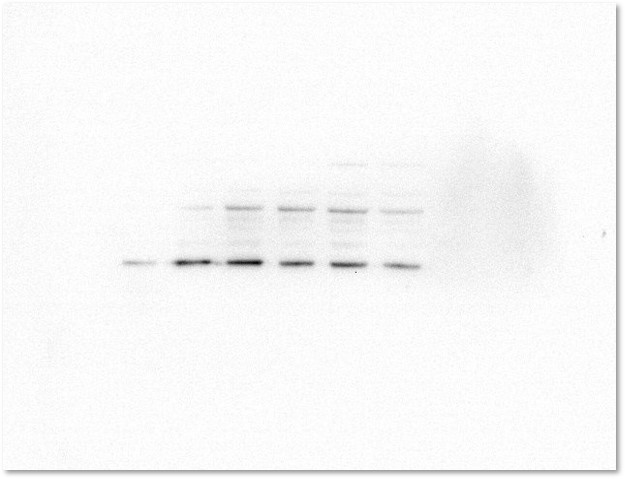

Supplement: Supplementary file 2 [file Supplementary_file_2.ZIP › supplement Figure/Figure 4. WBA p53_under lane.jpg]
